# Oncostatin M’s Involvement in the Pathogenesis of Chronic Rhinosinusitis: Focus on Type 1 and 2 Inflammation

**DOI:** 10.3390/biomedicines11123224

**Published:** 2023-12-05

**Authors:** Chie Ishikawa, Sachio Takeno, Yukako Okamoto, Tomohiro Kawasumi, Takashi Kakimoto, Kota Takemoto, Manabu Nishida, Takashi Ishino, Takao Hamamoto, Tsutomu Ueda, Akio Tanaka

**Affiliations:** 1 Department of Otorhinolaryngology, Head and Neck Surgery, Graduate School of Biomedical Sciences, Hiroshima University, Hiroshima 734-8551, Japan; chie0324@hiroshima-u.ac.jp (C.I.); kakomama@hiroshima-u.ac.jp (Y.O.); kwtm2022@hiroshima-u.ac.jp (T.K.); b203907@hiroshima-u.ac.jp (T.K.); kota61@hiroshima-u.ac.jp (K.T.); nm1027@hiroshima-u.ac.jp (M.N.); tishino@hiroshima-u.ac.jp (T.I.); takao0320@hiroshima-u.ac.jp (T.H.); uedatsu@hiroshima-u.ac.jp (T.U.); 2 Department of Dermatology, Graduate School of Biomedical Sciences, Hiroshima University, Hiroshima 734-8551, Japan; akiotan@hiroshima-u.ac.jp

**Keywords:** paranasal sinus, chronic rhinosinusitis, CRS, epithelial cell, eosinophil, oncostatin M, OSM, OSM receptor, OSMR

## Abstract

Objectives: The cytokine oncostatin M (OSM) elicits pathogenic effects involving disruption of the epithelial barrier function as a part of immunological response networks. It is unclear how these integrated cytokine signals influence inflammation and other physiological processes in the pathology of chronic rhinosinusitis (CRS). We investigated the expression and distribution of OSM and OSM receptor (OSMR) in CRS patients’ sinonasal specimens, and we compared the results with a panel of inflammatory cytokine levels and clinical features. Patients and Methods: We classified CRS patients as eosinophilic (ECRS, *n* = 36) or non-eosinophilic (non-ECRS, *n* = 35) based on the Japanese Epidemiological Survey of Refractory Eosinophilic Chronic Rhinosinusitis phenotypic criteria and compared their cases with those of 20 control subjects. We also examined OSM’s stimulatory effects on cytokine receptor expression levels using the human bronchial epithelium cell line BEAS-2B. Results: RT-PCR showed that the OSM mRNA levels were significantly increased in the CRS patients’ ethmoid sinus mucosa. The OSM mRNA levels were positively correlated with those of TNF-α, IL-1β, IL-13, and OSMR-β. In BEAS-2B cells, OSM treatment induced significant increases in the OSMRβ, IL-1R1, and IL-13Ra mRNA levels. Conclusions: OSM is involved in the pathogenesis of CRS in both type 1 and type 2 inflammation, suggesting the OSM signaling pathway as a potential therapeutic target for modulating epithelial stromal interactions.

## 1. Introduction

Chronic rhinosinusitis (CRS) is a disease characterized by symptomatic inflammation of the sinus mucosa lasting >12 weeks, as confirmed by endoscopy and/or imaging. CRS is classified into two types: type 2, associated mainly with a Th2 immune response, and non-type 2, characterized by a prevalence of type 1 and type 3 inflammation [1,2,3,4,5]. Eosinophilic chronic rhinosinusitis (ECRS) constitutes a subgroup of chronic rhinosinusitis with nasal polyps (CRSwNP) and is characterized by severe eosinophil infiltration. Pathological analyses of ECRS revealed a predominance of type 2 inflammation [3,6,7].

Oncostatin M (OSM) is a member of the interleukin (IL)-6 family of cytokines, which includes IL-11, IL-31, and leukemia inhibitory factor (LIF) [8]. OSM has been shown to be expressed by many cell types of the hematopoietic lineage, including T cells, neutrophils, macrophages, and eosinophils [9]. OSM is not known to be expressed by epithelium, fibroblasts, or smooth muscle, all of which express the two forms of the OSM receptor (OSMR) [10]. Individuals with CRS have been reported to have elevated levels of OSM in nasal polyp tissue, and OSM was shown to reduce the barrier function of the nasal mucosa [11]. Higher concentrations of OSM in sputum have been described in asthmatic patients with irreversible airflow obstruction [12,13]. In lung tissue, OSM has been found to increase airway hyper-responsiveness and cause eosinophilia [14]. Despite these intriguing findings, the precise mechanisms of OSM’s involvement in the development and progression of CRS remain unclear. To bridge this gap in knowledge, our study delved into the intricate relationship between OSM and cytokines, focusing on its association with type 1 and type 2 inflammatory processes.

The aim of this study was to improve our understanding of the molecular pathways involved in CRS and the role of OSM by identifying the complex interactions between OSM and various inflammatory mediators in CRS. Our findings pave the way for potential therapeutic interventions targeting OSM-related mechanisms. Through our comprehensive analyses, we hope to provide valuable insights into this field and, ultimately, advance the development of targeted therapies for patients suffering from this debilitating condition.

## 2. Patients and Methods

### 2.1. Study Design

This was a case-control study of 71 CRS patients who underwent endoscopic endonasal sinus surgery. The disease diagnosis was based on the patients’ history, clinical referred symptoms, endoscopic findings, and computed tomography (CT) images [6,7]. Patients with a previous sinus surgery were excluded. None of the patients had received systemic or topical steroids for ≥4 weeks prior to the surgery. The CT images were accessed by radiological grading using the Lund–Mackay system [15]. The presence of allergic rhinitis was diagnosed based on the patients’ clinical history, the presence of nasal symptoms together with positive nasal eosinophils, and positive allergen-specific IgE antibodies. We classified the CRS patients into ECRS and non-ECRS phenotypes based on the Japanese Epidemiological Survey of Refractory Eosinophilic Chronic Rhinosinusitis (JESREC) scoring system. The scores include four items: bilateral sinus disease with ethmoid sinus dominance, the presence of nasal polyps, the degree of eosinophilia in peripheral blood, and a mucosal eosinophil count ≥70/high-power field (HPF) [7]. Twenty patients without sinus infection who underwent endonasal surgery served as controls. All control subjects had normal-appearing paranasal sinus mucosa and normal radiological findings. The control subjects’ histories included surgery for nasal obstruction (*n* = 10), orbital decompression for dysthyroid ophthalmopathy (*n* = 4), endoscopic dacryocystorhinostomy (DCR) (*n* = 4), endonasal surgery for an intra-orbital tumor (*n* = 1), and fibrodysplasia ossificans progressiva (*n* = 1).

### 2.2. Quantitative RT-PCR Analysis

We obtained tissue samples from the ethmoid sinus, nasal polyps (if any), and the inferior turbinate at the time of surgery. When CRS findings were identified bilaterally, specimens were taken from both sides. We divided the surgical specimens and either immersed them in RNAlater™ solution (Thermo Fisher Scientific, Waltham, MA, USA) for reverse transcription-polymerase chain reaction (RT-PCR) or fixed them in 4% paraformaldehyde for immunohistochemistry. A quantitative PCR analysis was performed on an ABI Prism 7300 system (Applied Biosystems, Foster City, CA, USA), as described in [16]. Cellular RNA was isolated using RNeasy mini-kits (Qiagen, Valencia, CA, USA). Total RNA was then reverse transcribed to cDNA using a high-capacity RNA-to-cDNA kit (Applied Biosystems) according to the manufacturer’s instructions. Gene expressions were measured on a real-time PCR system using TaqMan Gene Expression Assays (Thermo Fisher Scientific). PCR primers that are specific for OSM (Hs00171165_m1), OSMRβ chain (Hs00384276_m1), tumor necrosis factor-alpha (TNF-α) (Hs99999043_m1), IL1-β (Hs01555410_m1), IL-1 receptor (Hs00991010_m1), IL-4 receptor-α (Hs00965056_m1), IL-13 (Hs00174379_m1), IL-13 receptor-α (Hs00609817_m1), and Gap43 (Hs00967138_m1) were used (Thermo Fisher Scientific). GAPDH (Hs02786624_g1) was used as a reference gene. Amplifications of the PCR products were quantified by the number of cycles, and the results were analyzed using the comparative cycle threshold (Ct) method (2^−ΔΔCt^). The target gene expression values are presented as relative ratios compared to the expression of the reference gene (ratio: target gene/GAPDH gene expression).

### 2.3. Immunohistochemistry

The primary antibodies used were anti-human OSMRβ mouse monoclonal antibody (#SC-271695; Santa Cruz Biotechnology, Dallas, TX, USA) and anti-human GAP43 rabbit polyclonal antibody (#16971-1-AP; Proteintech, Rosemont, IL, USA). Cryostat sections (approximately 5 μm-thick) were immersed in 3% H_2_O_2_ for 10 min for endogenous peroxidase deactivation and blocked with 10% goat serum in phosphate-buffered saline (PBS) containing 0.1% Tween 20 for 1 h at room temperature (RT). The slides were then incubated overnight at 4 °C with the primary antibodies. Staining and detection were performed according to the manufacturer’s instructions (Histofine Simple Stain kit; Nichirei Biosciences, Tokyo, Japan). Sections were counterstained with hematoxylin. Control specimens with IgG1 isotype control were used to verify that the nonspecific binding was not detectable. Consecutive sections were routinely stained with hematoxylin–eosin (HE) for the assessment of mucosal pathology and the degree of eosinophil infiltration.

### 2.4. Culture of Human Bronchial Epithelial Cells

Human bronchial epithelial cells (BEAS-2B) were purchased from KAC Co. (Kyoto, Japan). The cells were cultured in bronchial epithelial cell growth medium (BEGM™ BulletKit™; Lonza, Walkersville, MD, USA) under serum-free conditions in a 5% CO_2_ incubator at 37 °C. The medium was changed every 2–3 days. After the cells reached 80–90% confluence, subcultures were performed and then used for the experiments.

### 2.5. Data Analysis

The power and sample size calculations for the study design were performed based on data from a report of the OSM expression in CRS patients [11]. We used the G*power program ver. 3.1.9.6 for the estimation (https://www.psychologie.hhu.de/arbeitsgruppen/allgemeine-psychologie-und-arbeitspsychologie/gpower.html, accessed on 13 October 2023). For multiple comparisons, a screening of the data for differences was first carried out using the Kruskal–Wallis test. If the analysis yielded a significant result, a further comparison was performed by the Mann–Whitney U-test for the between-group analysis. Fisher’s exact test was used to compare qualitative data. Correlation coefficients were calculated by the Spearman method. Probability (*p*)-values < 0.05 were considered significant.

All procedures in this study complied with the ethical standards expressed in the Helsinki Declaration. The study protocol was approved by the Institutional Review Board of the Hiroshima University School of Medicine (Approval No. E2014-9136). Written informed consent was obtained from all patients prior to their participation.

## 3. Results

### 3.1. Background and Characteristics of the ECRS and Non-ECRS Subjects

The background and clinical characteristics of the study population are summarized in Table 1. We divided the 71 CRS patients into ECRS (*n* = 36) and non-ECRS (*n* = 35) groups based on the JESREC criteria. There was no significant age, gender, body mass index (BMI), or smoking history differences between the patient groups or between these two groups and the controls (*n* = 20). The ECRS group showed significant differences compared to the non-ECRS and control groups in the rate of asthma comorbid condition, blood eosinophil count, tissue eosinophil count, and CT score. The non-ECRS group showed significant differences in the tissue eosinophil count and CT score compared to the control group.

We also examined whether relationships existed between the cytokine expression levels and background factors that might act as confounding factors (Table 2). When we analyzed a population of all participants together, there were significantly increased OSMR and IL-13 mRNA levels in the male patients compared to the female patients. Interestingly, the participants with a BMI ≥ 25 showed significantly increased OSM, OSMR, TNF-α, and IL-1β mRNA levels compared to the patients with a BMI < 25. When the same analysis was carried out for each subgroup, the increase in OSM levels was significant only in the control group (*p* = 0.021). In the present study, there was no significant difference in cytokine levels between the age generations.

### 3.2. Target Gene Expressions in Sinonasal Mucosa

#### 3.2.1. Comparison of OSM and OSMR mRNA Expressions between the Controls and CRS Patients

**Figure 1:**
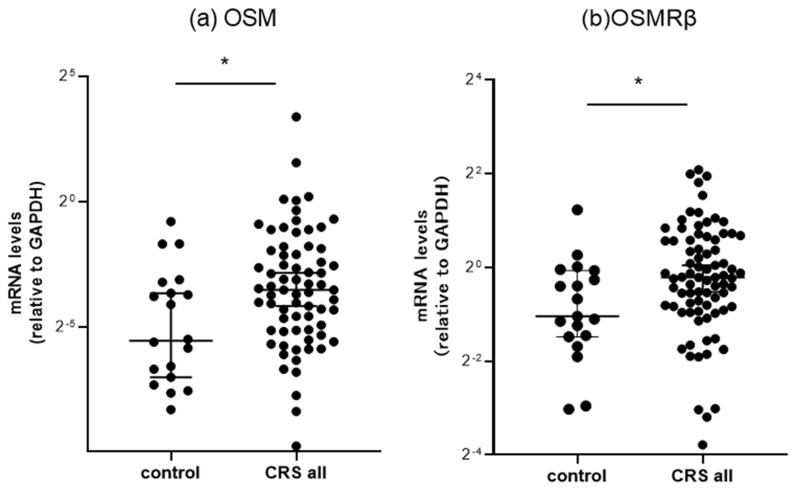
Comparison of mRNA expression in sinus mucosa of the controls and all CRS patients, detected by RT-PCR. The (**a**) OSM and (**b**) OSMRβ mRNA levels were quantitatively normalized against GAPDH levels. * *p* < 0.05. Center lines: median values, error bars: interquartile ranges, CRS: chronic rhinosinusitis.

We classified the ethmoid sinus mucosa and nasal polyp (NP) samples obtained during surgery into a control group and a CRS group, and we conducted an RT-PCR analysis to determine the mRNA expression levels of OSM and OSMRβ. The results showed that both OSM and OSMRβ transcripts were predominantly increased in both CRS groups (Figure 1a,b). A similar analysis was performed for the inferior turbinate mucosa, and no significant between-group differences were observed.

**Figure 2:**
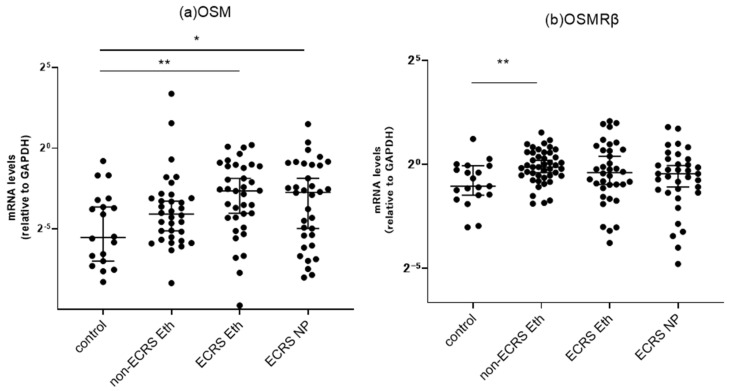
Comparison of the mRNA expression in paranasal sinus mucosa from the controls, non-ECRS patients, and ECRS patients, as detected by RT-PCR. The (**a**) OSM and (**b**) OSMRβ mRNA levels were quantitatively normalized against GAPDH levels. * *p* < 0.05, ** *p* < 0.01. Center lines: median values, error bars: interquartile ranges, ECRS: eosinophilic chronic rhinosinusitis, NP: nasal polyps.

We further subdivided the surgical specimens of the CRS patients into those of the non-ECRS and ECRS patients and compared the mRNA expression levels of OSM and OSMRβ based on phenotype differences. The results showed that OSM was predominantly increased at the ethmoid sinus mucosa and nasal polyps in the ECRS group, as compared to the controls (Figure 2a). The expression of OSMRβ also tended to be higher in the ECRS group, as compared to the controls; however, the differences were not statistically significant (*p* = 0.1717 for Eth and *p* = 0.3880 for NP). In contrast, there was a significant increase in OSMRβ mRNA in the ethmoid sinus mucosa of the non-ECRS group compared to the control group. OSM mRNA levels also tended to be higher in this group; however, no significant difference was observed between the non-ECRS and control groups (*p* = 0.1016 for Eth). The data on NP samples in the non-ECRS group are not presented because the number of samples was not large enough for a statistical analysis.

#### 3.2.2. Correlation with Inflammatory Cytokines

**Figure 3:**
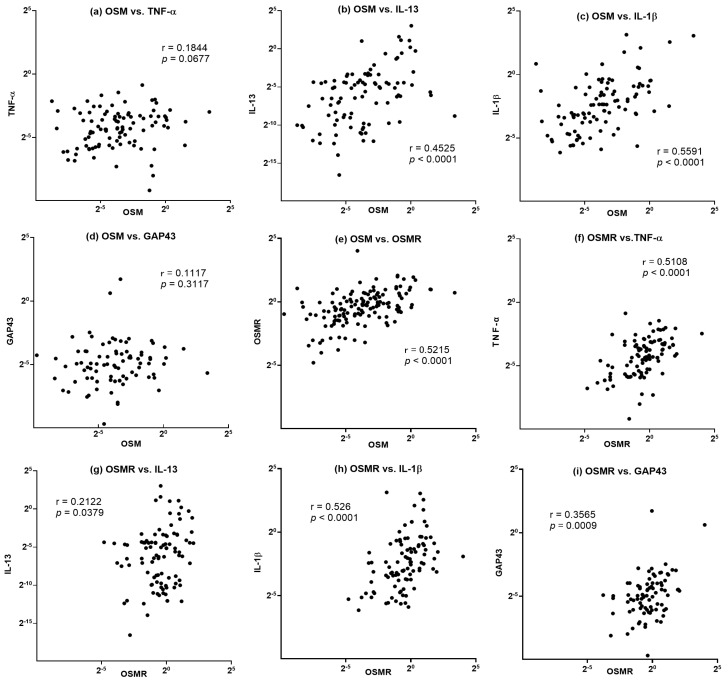
(**a**–**e**) Correlation of mRNA expression levels between OSM and a panel of inflammatory cytokines in sinus mucosa from CRS patients. (**f**–**i**) Correlations of mRNA expression levels between OSMRβ and a panel of inflammatory cytokines in sinus mucosa from CRS patients.

We further assessed the mRNA expression levels of TNF-α, IL-13, IL-1β, and GAP43 sampled from the same surgical specimens and conducted a correlation analysis to assess possible associations between these cytokines and OSM and OSMRβ (Figure 3). We observed an intimate positive correlation between OSM and OSMRβ (r = 0.5215). Additionally, OSM exhibited significant positive correlations with IL-1β (r = 0.5591) and IL-13 (r = 0.4525). As for OSMRβ, positive correlations were also observed with TNF-α (r = 0.5108) and IL-1β (r = 0.526).

### 3.3. Immunohistochemical Observation

**Figure 4:**
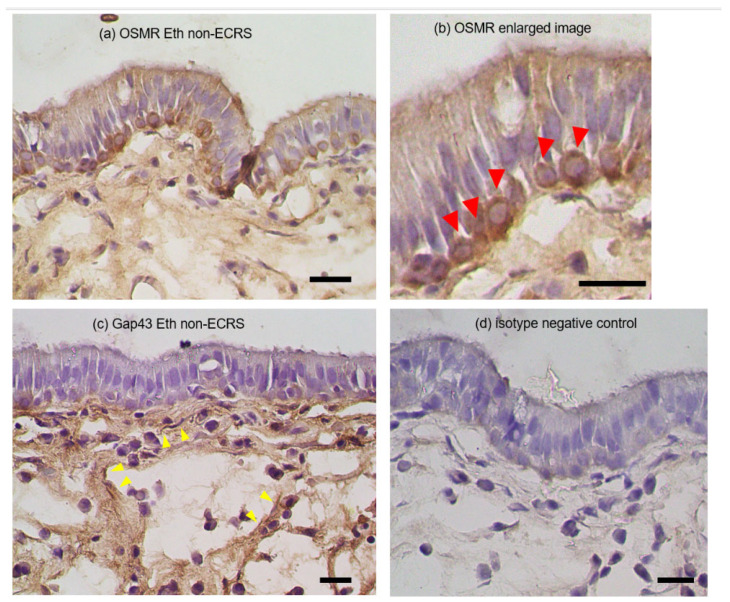
Representative immunohistological images showing the OSMR (**a**,**b**) and Gap43 (**c**) expression in ethmoid sinus mucosa sampled from CRS patients. Basal cell layers of ethmoid sinus epithelial cells and some mesenchymal cells in the submucosal layer stained positively for OSMR. Enlarged view of the intense cytoplasmic staining for OSMR in the basal cells ((**b**), red arrowheads). Positive Gap43 immunoreactivity was localized mainly with nerve bundle fibers in the submucosal area ((**c**), yellow arrowheads). Isotype negative control (**d**). Scale bars: 20 μm.

Since transcriptional changes in OSM were associated with CRS pathology and clinical manifestations, we next examined the sinus tissue distribution of OSMR and Gap43 proteins in representative cases. In the non-ECRS group, intense inflammatory cell infiltration with neutrophils and lymphocytes dominated the ethmoid mucosa on conventional histological examination. In contrast, dense eosinophil infiltration was observed in the ECRS group. Representative immunohistological images of the OSMR and Gap43 expression in ethmoid sinus mucosa sampled from CRS patients are provided in Figure 4. Basal cell layers of ethmoid sinus epithelial cells and some mesenchymal cells in the submucosal layer were stained positively for OSMR. Positive Gap43 immunoreactivity was localized mainly with nerve bundle fibers in the submucosal area.

### 3.4. Changes in Receptor Expression upon OSM Stimulation in Human Airway Epithelial Cells

**Figure 5:**
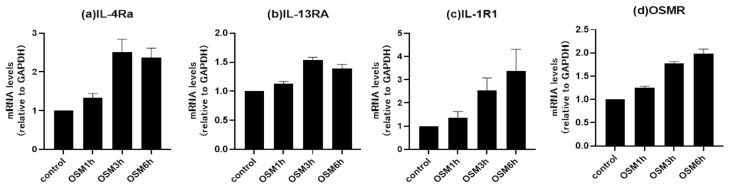
Time course changes shown by RT-PCR in the mRNA expressions of (**a**) IL-4Rα, (**b**) IL-13RA1, (**c**) IL-1R1, and (**d**) OSMRβ over time following treatment with 100 ng/mL of OSM.

Since IL-1β and IL-13 showed significantly positive correlations with OSM in sinonasal specimens, we next examined alterations in the cytokine receptor expressions after stimulating BEAS-2B cells with OSM at 100 ng/mL for various durations (pretreatment, 1 h, 3 h, and 6 h). The mRNA levels of IL-4Rα, IL-13RA1, IL-1R1, and OSMRβ were then measured by RT-PCR. The mRNA expression levels of all examined receptors exhibited a time-dependent increase when the human airway epithelial cells were stimulated with OSM (Figure 5).

## 4. Discussion

The expression levels of OSM and its receptor OSMRβ in the sinus mucosa of CRS patients were elevated compared to the control subjects, prompting us to explore these findings in the context of different pathological CRS conditions. We further assessed the possible relationship between OSM and various inflammatory cytokines, and we observed significant associations with type 1 and type 2 cytokines. CRS is categorized into type 2 CRS (which is associated primarily with the Th2 immune response) and non-type 2 CRS, characterized by the prevalence of type 1 and type 3 inflammation [1,2,3,4,5]. In type 1 inflammation, a hallmark cytokine, i.e., IFN-γ, produced by Th1 cells and innate lymphoid cells (ILC1), plays a key role. Type 2 inflammation involves cytokines IL-4, IL-5, and IL-13, produced by Th2 cells and ILC2 cells. Type 3 inflammation is associated with cytokines IL-17A and IL-22, produced by Th17 cells and ILC3 cells. In classical neutrophilic CRS, exposure to microbes, such as viruses and bacteria, induces type 1 and type 3 inflammation, i.e., non-type 2 inflammation. On the other hand, Th2 cells and ILC2 cells become activated in CRS with type 2 inflammation, as manifested by intractable eosinophilic inflammation with nasal polyp formation and increased mucin production, leading to the progression of refractory symptoms.

Eosinophilic chronic rhinosinusitis (ECRS) constitutes a subgroup within chronic rhinosinusitis with nasal polyps (CRSwNP) and is characterized by severe eosinophilic infiltration. This condition poses a significant challenge, as demonstrated by the findings of the JESREC Study [7]. Pathological analyses of ECRS revealed a predominant type 2 inflammation [3,6]. In contrast to non-ECRS, individuals with ECRS generally show resistance to conservative treatments, such as conventional macrolide therapy and mucolytic preparations. Endoscopic sinus surgery (ESS) is one option for disease management, combined with nasal irrigation and the use of oral corticosteroids. However, a considerable percentage of ECRS patients report the recurrence of symptoms with nasal polyp formation in the long term.

The aims of the present study to gain insights into the cellular response pathways triggered by OSM also meet clinical significance with the advent of the introduction of different biological agents. Biologics targeting type 2 inflammation have emerged, including omalizumab (an anti-IgE antibody), mepolizumab (an anti-IL-5 antibody), benralizumab (an anti-IL-5Rα antibody), and dupilumab (an anti-IL-4Rα antibody). All four of these agents demonstrated clinical efficacy in phase III trials for CRSwNP [17,18,19,20,21]. Among them, dupilumab binds specifically to the IL-4 receptor α subunit, shared by the IL-4 and IL-13 receptors, inhibiting signals from both IL-4 and IL-13. Dupilumab is a genetically engineered human IgG4 monoclonal antibody that broadly suppresses type 2 inflammation, offering the potential for improvement in pathological conditions [20]. Among the mentioned agents, only dupilumab is approved for use in ECRS patients in Japan and is available in clinical treatments.

As a member of the IL-6 family of cytokines, OSM exerts potent effects on stromal cell behavior in various tissues and organs. It is expressed and produced by multiple cell types within the hematopoietic lineage, including T cells, neutrophils, mast cells, macrophages, and eosinophils [9,22]. Human OSM signaling occurs through two receptors. The type I receptor is a heterodimer consisting of leukemia inhibitory factor receptor (LIFR) and gp130, and the type II receptor is a heterodimer composed of OSM receptor beta (OSMRβ) and gp130 [23]. In both scenarios, OSM initially binds to gp130 with low affinity, but effective signaling requires the subsequent recruitment of LIFR or OSMR, leading to the formation of high-affinity competent trimers [10]. In adult tissues, LIFR is expressed at low levels in a variety of epithelial, hematopoietic, and mesenchymal cell types and is not typically associated with pathological processes. In contrast, type II OSMR is highly expressed in numerous non-hematopoietic mesenchymal cells, including fibroblasts, endothelial cells, smooth-muscle cells, osteoblasts, and adipocytes. OSMR is also present in hepatocytes, mesothelial cells, glial cells, and epithelial cells in various organs [24,25]. Due to the fact that the expression of LIFR is not commonly linked with pathological processes, studies of OSM have often emphasized the crucial roles played by OSMR, rather than LIFR, in mediating OSM biology.

Increased OSM expression has been associated with inflammatory processes leading to barrier dysfunction in dermal and mucosal organs, such as the skin, lungs, and intestines. In the present study, we observed that the participants with a BMI ≥ 25 in the control group showed significantly increased OSM mRNA levels compared to those with a BMI < 25. It has been reported that OSM is also elevated in adipose tissue of patients with obesity and impaired glucose metabolism. OSM inhibits human adipogenesis, reduces glucose transporter type 4 (Glut4) expression, and induces an inflammatory state in human adipocytes [26]. OSM may be more likely to be elevated in sinus mucosa of obese patients with a non-diseased appearance. Elevated levels of OSM in nasal polyp tissues of patients with CRS have been encountered, and OSM has been reported to diminish the barrier function of the nasal mucosa [11]. Therapeutic interventions aimed at preventing barrier dysfunction or restoring the barrier once it is compromised could thus potentially be effective in treating inflammatory diseases in human airways, including asthma, CRS, and allergic rhinitis. Targeting OSM may prove beneficial in both non-ECRS and ECRS cases, in which the barrier dysfunction is sensitive to immune responses. As of this writing, no published study has explored the interplay between OSM function and the development of CRS with varying phenotypes in a Japanese population.

Our present analyses revealed that the levels of both OSM and OSMRβ were elevated in surgical specimens obtained from CRS patients. We further observed that when categorized by the disease groups, the OSMRβ level was predominantly increased in the non-ECRS group, and the OSM level was predominantly increased in the ECRS group. The results were partly consistent with those of an earlier report by Pothoven et al. [11]. They reported that OSM mRNA and protein levels were highly increased in nasal polyps obtained from the CRSwNP patients, as compared with those seen in control uncinate tissue (UT). They also found that OSMR mRNA expression was increased in nasal polyps and UT from patients with CRSwNP compared with that seen in control UT. The exact reasons for the different results remain unclear; however, it may reflect unidentical properties of harvested areas, i.e., Eth vs. UT, or different CRS etiologies between the United States and Japan. The definition of ECRS and CRSwNP is also different in the degree of eosinophilic infiltration, as reflected by genetic (ethnic) or environmental backgrounds.

We also clarified the sinus tissue distribution of OSMR proteins by immunohistochemistry in representative CRS cases. Predominant positive OSMR expression was located mostly in the basal cell layers of ethmoid sinus epithelial cells and some mesenchymal cells in the submucosal layer. These findings are consistent with the possibility that increased OSM expression triggered by inflammatory processes might play causative roles in barrier dysfunction in the epithelial layers in CRS patients with different phenotypes. However, we did not carry out a quantitative analysis to compare the expression levels among the ECRS, non-ECRS, and control groups. Further studies are warranted to evaluate the differences in the number of positive cells between different CRS phenotypes.

A significant correlation was also revealed between each cytokine and OSM, as well as OSMRβ. Notably, OSM levels were found to be positively correlated with the expressions of IL-1β and IL-13, indicating a potential association with type 1 and type 2 inflammation-related pathways. On the other hand, OSMRβ exhibited a significant correlation with TNF-α. These findings collectively suggest a complex interplay between OSM, OSMRβ, and the intricate network of cytokines involved in both type 1 and type 2 inflammation pathways. The elevated mRNA levels of OSM and OSMRβ in conjunction with these specific cytokines underscore the multifaceted nature of chronic rhinosinusitis, implicating OSM as a potential key player in the regulation of inflammatory processes. This intricate relationship opens new avenues for understanding the underlying mechanisms of CRS and holds promise for the development of targeted therapeutic strategies aimed at modulating OSM-related pathways in the treatment of this challenging condition.

GAP43 is a neuron-specific protein that is involved in the growth of nerve cells and axonal development [27,28]. We hypothesized that peripheral nerves in the nasal mucosa were undergoing proliferation in CRS, but the present results did not show any significant difference between the control group and the CRS group. In addition, there was no correlation with OSM levels. Immunofluorescence staining revealed the localization in areas presumed to be nerve fiber bundles in the submucosal region. Further research is needed to gain a better understanding of neurogenic inflammation in CRS.

An earlier study revealed that in the context of type 1 inflammation, OSM enhances the expression of OSMR and IL-1R1 in synovial fibroblasts, thereby amplifying the pathological effects of both OSM and IL-1 in rheumatoid arthritis (RA) [29]. In patients with RA, in line with these in vitro discoveries, it was reported that an intra-articular overexpression of OSM, along with TNF-α or OSM and IL-1β, leads to more extensive joint destruction compared to the impact of any single cytokine alone [30]. Regarding type 2 inflammation, heightened levels of OSM have been documented in sinus tissue from patients with allergic rhinitis and in the sputum of asthmatic individuals who exhibited irreversible airflow obstruction [12,13]. Elevated levels of OSM have also been observed in nasal polyps from individuals with CRS, as well as in tissue biopsies and induced sputum from persons with asthma. Biopsies from patients with eosinophilic esophagitis (EoE) showed increased OSM levels compared to controls [11,31]. In a mouse model, an intratracheal administration of an adenovirus that expressed OSM was shown to be sufficient to induce robust type 2 inflammation in the lungs, even without a specific antigen challenge [14].

Those findings indicated that OSM is involved in both type 1 and type 2 inflammation. In our present study, OSM showed correlations with cytokines related to both type 1 and type 2 inflammation. Moreover, we explored the impact of OSM on airway epithelial cells under inflammatory conditions, such as CRS. We measured the expression levels of cytokine receptors in order to understand the functional activation of these receptors in response to OSM. The findings revealed OSM involvement in both type 1 and type 2 inflammation. When we stimulated BEAS-2B cells with 100 ng/mL of OSM, we observed significant and temporal increases in the mRNA expression levels of IL-13RA1, IL-4Rα, and IL-1R1. These results provide insights into the cellular response pathways triggered by OSM by accessing receptor expression levels. The elucidation of downstream signaling events inside cells initiated by cytokine receptor activation is crucial, as it provides valuable information on the signaling cascades triggered by OSM and its interaction with specific receptors. We consider these analyses essential for comprehending the complex cellular responses related to CRS.

As our experiments in this study focused mainly on type 1 and type 2 inflammation, we did not examine the association between the cytokines associated with type 3 inflammation and OSM. This is a subject for future work.

In summary, our results suggested that OSM levels are increased in the nasal mucosa of CRS patients and are associated with both type 1 and type 2 inflammation. OSM was also reported to disrupt nasal mucosal barrier function [11]. In light of these findings, it is possible that OSM is multi-factorially involved in the pathogenesis of CRS. Notably, the anti-OSMRβ antibody vixarelimab (KPL-716) is currently undergoing clinical trials as a treatment for nodular prurigo (ClinicalTrials.gov Identifier: NCT03816891) [32]. As our understanding of OSM’s role in the pathogenesis of CRS deepens, there is optimism for the development of new therapeutic options for this prevalent disorder.

## Figures and Tables

**Table 1:** Background and clinical characteristics of the study population.

	Controls	Non-ECRS	ECRS
*n* (male/female)	20 (6/14)	35 (21/14)	36 (18/18)
Age, mean ± SD	49.6 ± 15.6	51.5 ± 15.4	54.5 ± 10.2
Allergic rhinitis, %	8 (53.3%)	25 (71.4%)	27 (75%)
BMI, kg/mm^2^, mean ± SD	22.4 ± 4.2	23.3 ± 3.8	22.6 ± 3.6
Bronchial asthma, %	1 (5%)	5 (14.3%)	17 (47.2%) **^,†††^
Blood eosinophils, %; median, range	2.5 (0.0–6.6)	1.6 (0.0–20.0)	6.8 (1.1–13.9) ****^,†††^
Tissue eosinophils, cells/HPF; median, range	4.97 (0.0–23.0)	7.3 (0.0–70.3) ^††^	117.0 (4.0–383.3) ****^,†††^
CT score, mean ± SD	2.45 ± 4.24	7.7 ± 5.2 ^†††^	14.8 ± 5.0 ****^,†††^

Data are mean ± standard deviation (SD), median (range), or number (%). ** *p* < 0.01, **** *p* < 0.0001 vs. the other groups. ^††^ *p* < 0.01, ^†††^ *p* < 0.001 vs. the control. BMI: body mass index, ECRS: eosinophilic chronic rhinosinusitis, HPF: high-power field (400×), CT: computed tomography.

**Table 2:** Cytokine levels in relation to clinical background factors for all participants.

	*n*	OSM			OSMR			GAP43		
		Median	IQR	*p*	Median	IQR	*p*	Median	IQR	*p*
Male	45	0.0937	0.3953	0.3613	0.8850	1.0475	0.0398 *	0.0263	0.0355	0.0500
Female	46	0.0769	0.1554		0.6654	0.7657		0.0416	0.0435	
With AR	60	0.1013	0.2456	0.3263	0.8423	0.9861	0.3221	0.0269	0.0343	0.0088 **
Without AR	31	0.0732	0.2397		0.7572	0.6298		0.0531	0.0808	
With BA	23	0.1614	0.4173	0.0720	0.8926	1.0689	0.2261	0.0421	0.0336	0.7106
Without BA	68	0.0730	0.1853		0.7572	0.7503		0.0304	0.0505	
BMI ≥ 25	21	0.1919	0.4764	0.0168 *	1.0320	1.1745	0.0218 *	0.0325	0.0561	0.8702
BMI < 25	70	0.0698	0.1547		0.7324	0.7393		0.0329	0.0469	
	***n***	**TNF-α**			**IL-13**			**IL-1β**		
		**Median**	**IQR**	***p***	**Median**	**IQR**	***p***	**Median**	**IQR**	***p***
Male	45	0.0692	0.0876	0.1436	0.0447	0.1142	0.012 *	0.1872	0.4529	0.4139
Female	46	0.0488	0.0806		0.0129	0.0418		0.1365	0.3718	
With AR	60	0.0511	0.0829	0.1435	0.0283	0.0686	0.8400	0.1705	0.2966	0.4715
Without AR	31	0.0742	0.0835		0.0141	0.0472		0.2321	0.5690	
With BA	23	0.0508	0.0766	0.3823	0.0373	0.0726	0.3775	0.2439	0.4067	0.5465
Without BA	68	0.0543	0.0995		0.0148	0.0543		0.1247	0.3906	
BMI ≥ 25	21	0.0904	0.0822	0.0291 *	0.0364	0.3174	0.2511	0.3406	0.9144	0.0155 *
BMI < 25	70	0.0498	0.0845		0.0195	0.0470		0.1238	0.3009	

Data are median and interquartile ranges. * *p* < 0.05, ** *p* < 0.01 vs. the other groups. AR: allergic rhinitis, BA: bronchial asthma, BMI: body mass index.

## Data Availability

The data presented in this study are available upon reasonable request from the corresponding author.
